# Trachoma and its determinants in Mojo and Lume districts of Ethiopia

**Published:** 2012-12-25

**Authors:** Kassahun Negash Yalew, Medhanit Getachew Mekonnen, Atsbha Asrat Jemaneh

**Affiliations:** 1 Africa Medical and Research Foundation (AMREF), Ethiopia

**Keywords:** Trachoma, risk factors, eye diseases, Ethiopia, blindness

## Abstract

**Introduction:**

Trachoma is a public health problem in Ethiopia accounting for 35–50% of cases of blindness. This study aimed to determine the prevalence of trachoma and its determinant factors in Mojo and Lume districts.

**Methods:**

A cross sectional community-based survey was conducted. From the two districts, a total of 23 clusters were selected by a multistage cluster random sampling technique. A total of 731 households were visited using structured questionnaires and clinical manifestation of trachoma was examined by ophthalmic nurses to assess stages of trachoma in children between ages 1 and 9 years and adults aged above 15 years.

**Results:**

Among 431 examined children, 54(12.53%) had trachomatous inflammation-follicular (TF) and 43(9.98%) had trachomatous inflammation-intense. Among the adults we found 12 (1.68%) prevalence of trachomatous trichiasis. The presence of latrine (p=0.02), garbage disposal system (p=0.05), main source of water consumption (p=0.01) and keeping animals in the living room were found to be significant risk factors (p<0.001).

**Conclusion:**

Prevalence of trachoma was found to be 12% which is higher than the WHO standard. The study also identified that there was significant association between the different stages of trachoma with risk factors such as source of water and keeping animals in the living room.

## Introduction

Visual impairment and blindness have been recognized worldwide as one of the major public health problems, especially in developing countries where most of the blind live and resources are minimal. According to WHO, globally about 37 million people are blind and 124 million people have low vision. Internationally, about a quarter of blindness is avoidable and is mainly caused by cataract and trachoma [1]. International actions to prevent avoidable blindness have been gaining attention over the recent decades. Vision 2020: “The Right to sight” is a global initiative launched in 1999 with the objective of eliminating the main causes of avoidable blindness by the year 2020 [1].

Trachoma is a bacterial illness caused by Chlamydia trachomatis. Typically this disease is associated with environmental factors and poor hygiene and is transmitted through contact with infected individual, contaminated water, or through flies that have come into contact with infected materials. Once an individual has been reinfected multiple times, the inside of the eyelid becomes scarred, forcing the eyelid to invert. The position of the eyelid causes the infected individual's eyelashes to scratch the cornea, causing permanent visual damage.

The burden of blindness in the Sub-Saharan Africa is the greatest of all the other regions of the world. Sub-Saharan Africa contains less than 10% of the world's people, but 20% of the world's blind. In contrast, countries with established market economies account for 15% of the global population, but only 6% of blindness [2]. The prevalence of blindness is 10-20 times greater in the developing countries than the developed countries. One country that possesses high rates of vision impairment is Ethiopia. Ethiopia is believed to have one of the world's highest rates of blindness and low vision. According to the Prevalence of Blindness, Low Vision, and Trachoma in Ethiopia from the total 75 million population, 1.2 million are blind, 2.8 million people have low vision, 9 million children of the age group 1-9 years have active trachoma, and 1.3 million adults live with trachomatous trichiasis (TT) [3]. More than a quarter of blindness in Ethiopia is either preventable or curable. According to the National Survey on Blindness, prevalence of blindness in Ethiopia is 1.6% (1.1% for urban and 1.6% for rural population) and low vision is 3.7% (2.6% for urban and 3.8% for rural). Blindness and low vision are more prevalent among females; 1.9% versus 1.2% for blindness, and 4.1% versus 3.1% for low vision. Prevalence of childhood blindness is 0.1% and accounts for 6% of the total blindness burden in Ethiopia [3].The major causes of blindness in Ethiopia are cataract (49.9%), trachomatous corneal opacity (11.5%), refractive error (7.8%), other corneal opacity (7.7%), glaucoma (5.2%) and macular degeneration (4.6%) [3]. The national prevalence of active trachoma either TF/TI for children in the age group 1-9 year is 40.1%. There is significant regional variation of active trachoma prevalence with the highest prevalence being in Amhara (5.2%). The national prevalence of trachomatous trichiasis (TT) for age group 15 and above in Ethiopia is 3.1%. Trachomatous trichiasis is highest in females compared to males (4.1% versus 1.6%). The prevalence of trachoma in Eastern Showa is not well known. This research was done to assess the prevalence of trachoma and its determinant factors in Lemu and Mojo districts.

## Methods

### Study Setting

East Shewa is one of the 28 administrative zones in the Oromia Region, located at the South-eastern escarpment with an estimated population of 1,278,362 in 2006/2007. The capital of the zone, Adama, is located at a distance of about 100 km east of Addis Ababa, on the main way to Harar and Dire Dawa in eastern Ethiopia. The zone has 12 districts, most of which are situated in the Great East African Rift Valley lakes region that crosses the country. In terms of ethnicity, the East Shewa Zone communities belong mainly to the Oromo ethnic group while more heterogeneous within the urban centers.

This study was conducted in two districts of the Oromiya Region, Lume and Mojo districts. According to, the 2007 National Census Report the districts have a total population of 117,080, and 450,412 respectively.

### Study population

The population that was studied comprised children between one and nine years of age as well as adults older than 15 years.

### Sample size calculation and sampling

A sample size estimated using a standard cross-sectional proportion sample size calculation formula is implemented. The expected prevalence of TF in conducted studies by Yemane was 41.4% [4]. Assuming that risk of the true prevalence was outside the confidence interval (5%), an alpha risk of 5%, and a z-score of 1.96; then the calculated sample size was 750.

### Primary sampling unit

The primary sampling unit (PSU) for the survey was a kebele; each kebele is regarded as a cluster. At the districts level twenty clusters were selected, proportional to their size. The list of current kebeles in each district was obtained from the local administration. Then the proportion of children under 9 years was statistically interpolated. The following assumptions were adopted: 32% of the general population composed of those less than five years old and each household on average is comprised of five individuals. This figure was taken from the household distribution described in the Ethiopian Demographic and Health Survey.

Kebeles that were not reached within half day walking from the nearest driving point were regarded as geographically inaccessible. Clusters inaccessible, due to insecurity or geographical barriers, were excluded from the survey prior to the selection of the clusters.

### Data Analysis

During the data collection, supervisors watched over the data collectors on site and every evening checked the data for accuracy, consistency and completeness. The data was entered and analyzed using SPSS software (Version 19). The association between the risk factors and the different stages of trachoma were analyzed using Pearson's Chi-square test. Logistic regression analysis was used to identify the determinant factors among the associated variables.

## Results

### Survey Description

From the two districts a total of 23 clusters/ kebeles were randomly selected; 5 clusters from Mojo and 18 clusters from Lume Districts. A total of 713 households were visited and clinically examined by Ophthalmic Nurses. 64% (454) of the total visited households had children in the age range 1-9 years and 431 all of them were selected for clinical examination; 79(18%) and 352(81%) from Mojo and Lume districts respectively. The distribution of household members by clusters and districts are presented in Table 1.

**Table 1:** Characteristics of the respondents

Characteristic	District				Total	
	Mojo		Lume			
	Number	%	Number	%	Number	%
**Sex of the respondent**						
Male	35	23	196	35	231	32
Female	115	77	367	65	482	68
**Age group**						
10-24	29	20	105	18	134	19
25-40	75	49	274	49	349	49
> 40	46	30	184	33	230	32
**Status of the respondent in the HH**						
Head of the HH	38	25	189	34	227	31.8
Wife	95	64	311	55	406	56.9
Son/daughter	17	11	63	11	80	11.2
**Religion**						
Orthodox	130	86	542	96	672	94.2
Muslim	7	5	7	1	14	2.0
Protestant	13	9	13	2	26	3.6
Other	0	0	1	0	1	0.1
**Educational Status**						
Illiterate	41	27	339	60	380	53.3
Can read and write	3	2	29	5	32	4.5
1-4 grade completed	22	16	46	8	68	9.5
5-8 grade completed	35	23	100	18	135	18.9
9-12 grade completed	40	27	45	8	85	11.9
College education	9	6	4	1	13	1.8
**Occupation**						
Farmer	7	5	405	70	412	57.8
Daily laborer	41	27	72	13	113	15.8
Government employee	40	27	12	2	52	7.3
House wife	27	18	25	4	52	7.3
Merchant	19	13	41	7	60	8.4
Others	16	11	8	1	24	3.4

As portrayed in summary Table 1, most of the clusters/ kebeles had 4-6 members in their house hold. In general, 90(58%) of households had 4-6 members, 43(29%) 1-3 members and 17(11%) 7-9 members in their house.

Table 2 indicates that most of the clusters in Lume district had 4-6 family members. There were also Ketenas that had significant members (7-9); these included Kurma Fatolla, Tilty Gerbi and Tulured. On the other hand, Kunche and Ejersa Jorro among the ketanas with lower percentage contributors for the total family members of high family size categories i.e. 7-9 members compared to other ketanas. Out of the total surveyed household 284(50%) had 4-6 members 162(29%), 1-3 members and the remaining 21% had 7-9 members in their household.

**Table 2:** Main source of water for the Household and estimated time taken

	District				Total	
	Mojo		Lume			
	Number	%	Number	%	Number	%
**Source of water**						
Pipe	149	99	492	87	641	90
Protected well	1	1	46	8	47	7
Unprotected well	0	0	1	0	1	0.1
River	0	0	6	1	6	0.8
Pond/surface water	0	0	13	2	13	1.8
Others	0	0	5	1	5	0.7
**Time taken to the source of water**						
In compound	94	59	75	13	169	24
<30mins	46	33	293	51	339	48
30-59mins	9	7	164	29	173	24
60-89mins	0	0	20	3	20	3
2-4 hours	1	1	11	2	12	2

### Household Characteristics

A total of 713 households were visited, 80% of them were enumerated in Lume district. The characteristics of the study participants of the household are presented below. Table 1 shows the demographic characteristics of respondents. Of the total 713 respondents, 68% were females and the remaining 32% were males. The age distribution of the population surveyed showed that 19% of respondents were 10-24 years and the largest proportion of respondents (i.e. 49%) were 25-40 years old while the remaining population was above 40 years old. Among the individuals that participated in the survey, 227(31.8%) reported being the head of the household and 406(56.9%) of the respondents were house wives and 80(11.2%) participants were sons or daughters within the household.

House wives are the majority within the Lume District. 189(34%) of respondents reported being the head of a household while 63(11%) claimed to be a son/daughter. Majority of respondents in both Lume and Mojo districts were house wife respondents; 311(55%) and 95(64%) respectively. When analyzing religious backgrounds of participants, approximately 94.2% of the respondents were Orthodox Christian in both districts while the remaining 5.7% represented other religious followers.

Educational status of the respondents showed that 53% were illiterate, mostly within the Lume district (60%). These results showed that households in the urban areas, specifically in the Mojo district, are relatively more literate than those found in the rural areas. In the Mojo district, further analysis indicated that 23% and 27% of respondents completed primary school (grade 5-8) and high school respectively. Overall, the occupational status of the respondents revealed that 57.8% were farmers, while daily laborer, merchants, government employees and house wives were reported by the respondents according to their significance.

### Main source of water and accessibility of water supply

As seen in Table 2, a majority of households obtained water primarily from pipes. However the average travel time to get this water source was less than 30 minutes. Moreover, more than 24% of respondents reported that they traveled 30 to 60 minutes to a water source. Pipe water is the main source of water for urban (Mojo) households (99%) while protected well water source is common in Lume (87%).

A majority of the households observed (90%) get drinking water from pipelines, followed by 7% from protected well and 0.1% from unprotected wells. Approximately 48% of the surveyed households traveled less than 30 minutes range of distance to use water for daily consumption. The availability of water in the compound was significantly higher in Mojo town (59%) compared to 13% in Lume district.

### Sanitation status of the surveyed household

Based on Table 3, 32% of respondents kept their animals in the range of 1-3 meters of their house while 21% of animals were kept in the range of 4-6 meters. In general, 56% of the respondents (13% Mojo; 62% Lume) kept animals within range of less than 7 meters radius of their house. 68% kept animals separately from their living room in both district, 30% kept animals in their living room only at night and 15% kept animals in their living room for both day and night; most of them were from Mojo district. A majority of respondents (63.9%) primarily disposed of their garbage in open fields, followed by 29.2% of residents in both districts disposed of garbage in an uncovered pit.

**Table 3:** Sanitation Status of Surveyed Household by District

	District				Total	
	Mojo		Lume			
	Number	%	Number	%	Number	%
**Animals kept within 20m of the respondent house hold**						
No	132	87	184	34	316	44.3
Yes, 1-3m	10	7	216	38	226	31.7
Yes, 4-6m	8	6	138	24	146	20.5
Yes, 7m or more	0	0	25	4	25	3.5
**Animals kept in the housegold of the respondent**						
No, keep separately	16	68	293	68	309	68.1
Yes, only night	2	9	134	31	136	30.0
Yes, only day time	0	0	2	0	2	0.4
Yes, both at night and day	5	23	2	0	7	1.5
**Garbage disposal place**						
In open field	54	37	397	71	451	63.9
Uncovered pit	11	8	10	2	21	3.0
Covered pit	56	38	150	27	206	29.2
Other	26	18	2	7	28	4.0
**Access to latrine**						
No, use open field	11	7	156	28	167	23.4
Yes, covered pit latrine	72	48	174	31	246	34.5
Yes, uncovered pit latrine	67	45	232	41	299	41.9
Yes, but not used currently	0	0	1	0	1	1
**Who in the household, regularly use latrine**						
Only adult	55	39	143	34	198	35
Only children	85	61	275	66	360	64
Both adult and children	0	0	1	0	1	0

Latrines were classified as covered pit latrines, uncovered pit latrines and open fields. A significant proportion of households reported that they had no access to latrine service and they used open fields 167(23.4%) while a considerable proportion (i.e. 299 (42%)) of households used uncovered pit latrine for human excreta disposal. Only 246 (35%) of the total respondents have reported that they had access to covered pit latrine. Almost two thirds, 360(64%) regular users of the covered pit latrines among the household members were children.

### Face washing habit and facial cleanness

The face washing habit and facial cleanliness of Mojo and Lume districts is presented in Table 4. The frequency of face washing habit in both districts showed that 47%, 30% and 17% of respondents washed their face twice, three times and once in a day, respectively. Overall there is a similarity in the frequency in face washing practices in the two districts except there is a significant variation in practice of washing their face once a day in Lume than Mojo district.

**Table 4:** Face washing habit and facial cleanness by District (not all responded)

	District				Total	
	Mojo		Lume			
	Number	%	Number	%	No	%
**Frequency of face washing**						
Once a day	7	6	102	20	109	17
Twice a day	54	42	245	48	299	47
Three times a day	43	34	148	29	191	30
Once a week	1	0.8	2	0.4	3	0
Others	23	18	10	2	33	5
**Children use soap**						
Yes	95	89	402	81	497	83
No	12	11	93	19	105	17
**Why wash their face more than once**						
The area is dusty	31	36	171	51	202	48
Believe we can prevent diseases	54	64	165	49	219	52

It is also observed that the majority of children (83%) have used soap to wash their face. The results also showed that the practice of children washing their face using soap between the two districts is almost similar. Asking the respondents about the reason for practicing face washing frequently showed that 52% answered that it was to to prevent disease while 48% revealed that it was to clean their face from dust. More urban dwellers believed that frequency of washing prevents disease than the rural dwellers.

### Perceptions on trachoma as a major health problem

Table 5 shows the types of health problems, source of information delivered, and mechanisms of transmission between the two woredas. The distribution of the problem between the two woredas is more or less similar though there is a significant variation in the distribution of other health problems. Slightly more than a quarter, 181(26%) of the respondents believed that trachoma is a major health problem of the districts followed by malaria with 192(22%). On the contrary, significant respondents 408(58%) mentioned other health problems as major rather than the five mentioned health problems in the study.

**Table 5:** Health problem by district and source of information on Trachoma and mechanisms of transmission

	District				Total	
	Mojo		Lume			
	Number	%	Number	%	Number	%
**Major health problem in the area**						
Malaria	7	4	185	34	192	22
Tuberculosis	1	1	8	1	9	1
Trachoma	35	24	146	27	181	26
Diarrhea	7	4	56	10	63	9
HIV/AIDS	11	7	5	1	16	2
Others	111	75	297	54	252	58
**Source of information for Trachoma**						
Health Worker	54	40	146	31	200	33
Health Extension Worker	19	14	140	30	159	26
Media	56	41	181	39	237	39
Family members	34	25	59	12	93	15
Local leaders	0	0	6	1	6	1
**Mechanisms of Trachoma transmission**						
Flies	97	71	318	68	415	69
Poor personal hygiene	79	58	194	41	273	45
Poor environmental condition	28	21	89	19	117	19
Other	4	3	6	1	10	2

The main mentioned sources of information about trachoma were media 237(39%), health worker 200(33%) and health extension worker with 26%. The distribution between the two districts is almost similar concerning media as a source of information while the other source of information showed slight variations between the districts. The primary transmission of trachoma is through flies according to the majority of the respondents (69%). The second major mechanism of transmission of trachoma mentioned by the respondents was poor personal hygiene 273(45%) followed by poor environmental condition 117(19%).

Major risk factors such as the availability of latrines, garbage disposal mechanisms, and the main water sources were assessed using a Chi-square test among children and adults respectively. The prevalence of TF and TI in the woredas was tested using a p-value =0.05 and the results showed that the rate of occurrence of TF and TI among children between 1-9 years old showed significant association among the following risk factor of availability of latrine, garbage disposal and keeping animals in the living room.

### Prevalence and Determinant Factors of Trachoma

From 431 selected children for clinical examination, 54(12.53%) and 43(9.98%) of them were affected by TF and TI respectively. The prevalence of the different stages of trachoma (TS, TI and CO) among adults and the risk factors reported in the study showed that age was a factor which significantly affected the level of manifestation resulting in a p-value =0.01. Availability of latrine and TS stage of trachoma also had significant p-value of less than 0.05 while the remaining stages of trachoma (TI and CO) were insignificantly associated with the availability of latrines as a risk factor. Keeping animals in the main living room also has a significant association with the prevalence of TS and TI with significant p-values of .01 and .05, respectively. In general, 12(1.68%) of the respondents had contracted trichiasis and 4(0.6%) manifested corneal opacity (CO).

The other risk factor for the prevalence of trachoma (TI, TS and CO) identified in the study was the source used to obtain water. The p-value for TI, TS, and CO are 0.04, 0.02 and <0.001 respectively meaning that source of water was a significant factor with strong association to the three mentioned stages of trachoma. The overall rate of households who had TT surgery was lower (4%) in the districts. The distribution of TT surgery in the two woredas showed that in the rural areas (5%) there are higher surgery rates than the urban regions (0.7%). Among the people who have undertaken TT surgeries were 15 (50%). Fourteen (47%) of them reported positive sentiments concerning the surgeries, respectively while only 3% (1 person) of people were disappointed by the surgery. The majority of the respondents (90% (28)) received TT surgery in health facilities while only 10% (3) of people underwent surgery in a health post.

Table 6 shows facial cleanliness of adults by districts. Of the total number of households 66(9%) of members’ faces were unclean while 647(91%) cleaned their faces. The number of households who used azithromycin was analyzed, in which the frequency of azithromycin usage and the reason for usage of azithromycin were examined. Of the total respondents, 620(87%) of the households took azithromycin twice a day (70%) and 30% of azithromycin users only took the medication once a day. The majority of respondents (68%) mentioned the lack of the availability of azithromycin as a reason for only using it once in a day while 18% mentioned painful side effects associated with taking azithromycin. Fear of side effects was another reason for those who refused to use azithromycin and the major fear associated with those who did not take the medication was diarrhea (83%) and abdominal cramps 45%.

**Table 6:** Distribution of the households taking azithromycin and frequency

	District				Total	
	Mojo		Lume			
	Number	%	Number	%	Number	%
**Number of Household taking azithromycin**	123	82	497	88	620	87
Frequency of azithromycin						
Only once	26	19	194	33	220	30
Twice	111	81	398	67	509	70
**Reasons for the Household member taking only once**						
Painful	0	0	39	21	39	18
They were not around	28	93	116	62	144	68
Pregnancy	2	7	13	7	15	7
Others	0	0	23	12	23	11
**Reasons not taking azithromycin, fear of side effect**						
Nausea	0	0	7	9	7	7
Diarrhoea	5	38	39	48	44	46
Vomiting	1	10	0	0	1	1
Abdominal Cramp	8	52	35	43	43	45
Others	0	0	1	1	1	1

## Discussion

Trachoma is a communicable disease attributed to poor personal hygiene, environmental sanitation, and a lack of adequate and clean water supply. If left untreated, blindness can occur. The major causes of blindness are cataract (49.9%) and trachomatous corneal opacity (11.5%) [3].

According to this study conducted in the Lume and Mojo districts, the prevalence of TF for children between 1-9 years old is 13% which is much lower than the national prevalence of 40.1% [4]. In this same study trachomatous trichiasis (TT) in adults showed a much lower prevalence than the national standard (3.1% vs 1.68). However, the current prevalence is still higher compared to the WHO standard (<1% for TT) [1]. Trachoma is highly correlated with the availability of water and behavior towards face washing habit in the community. About six in every ten (62.4%) of the children had a clean face on examination; 72.5% households reported washing faces of children two or more times a day. This study showed that 77% of the respondents have a habit of washing their face 1-2 times a day [5].

More than half of the respondents in the study area keep animals less than 7m in the living room. Different literature stated that flies breed in and are likely to be attracted to rubbish dumps, bins, excreta including cow dung, decaying food and rotting carcasses. In this study, keeping animals in the house significantly related with the number of trachoma in the household and in the community in general. This may be related the animal dung to be a breeding site for the flies and also increases the exposure of children to the flies. Flies are attracted to red eyes with discharge, and carry the organism Chlamydia trachomatis to the eyes of others within a family or a community, both in children and adults [6].

Frontline health personnel can play key role in preventing and controlling blindness if they have good understanding of primary eye care (PEC) [8]. However, this research showed that 21% of the population has the opportunity to get information about trachoma in the study area. It is believed that health extension workers (HEW) can play a significant role in primary eye care if they are adequately trained and well motivated. However, there is little information on how HEWs can participate in community level promotion of eye health in Ethiopia.

Most medications that are taken come with some type of side effect. Some side effects may be greater than others, but all should be taken seriously. This study showed that about 18% of respondents are not willing to take azithromycin to prevent trachoma attributed to the fear of side effect.

## Conclusion

Prevalence of trachoma manifested among the interviewed and clinically examined children and adults was found to be 12% and 1.68% respectively; figures that are higher than the WHO standard of less than 1%. It was also observed that the association between the different stages of trachoma with risk factor such as keeping animals in the living room and source of water was significant. There is need to develop a strategy to strengthen community based information and education through the existing health extension workers about trachoma. Primary school has to be a target place to educate children on personal and environmental hygiene particularly facial cleanness. Proper education and awareness has to be raised with regard to the misconception on zitromax side effect.Prevalence of trachoma manifested among the interviewed and clinically examined children and adults was found to be 12% and 1.68% respectively; figures that are higher than the WHO standard of less than 1%. It was also observed that the association between the different stages of trachoma with risk factor such as keeping animals in the living room and source of water was significant. There is need to develop a strategy to strengthen community based information and education through the existing health extension workers about trachoma. Primary school has to be a target place to educate children on personal and environmental hygiene particularly facial cleanness. Proper education and awareness has to be raised with regard to the misconception on zitromax side effect.
